# A Book Review on: Sherris Medical Microbiology – International Edition, 6th Edition

**DOI:** 10.3389/fcimb.2015.00034

**Published:** 2015-04-10

**Authors:** Erlangga Yusuf

**Affiliations:** Department of Medical Microbiology and Infection Control, UZ BrusselBrussels, Belgium

**Keywords:** book review, textbook, medical microbiology, infection, medical students

The sixth edition of Sherris Medical Microbiology appearing 5 years after the previous edition provides brief reviews of major pathogens and the associated infectious diseases. This book can be used as a framework to organize knowledge on the diagnostics of infectious diseases that can be easily found on internet nowadays. It appears to be designed for medical students and medical microbiology residents, but medical microbiologists, infectiologists, or general internists are also potential readers who can use this book for rapid review.

The editors have assembled a limited number of authors (six) and uniform chapter writing can be expected. Every chapter on specific pathogens commences with microbiological aspects of the pathogens followed by the disease epidemiology and pathogenesis, and clinical aspects (manifestations, diagnosis, and treatment and prevention). Some chapters are relatively exhaustive compared with others, for example the chapter on influenza and parainfluenzae virus, due to great clinical and epidemiologic importance. This book adds special features such as margin notes that highlight important points and “clinical capsule” that cover the essence of the disease caused by the pathogens. The tables in this book can be used for quick review by a beginner in medical microbiology, for example an overview of human microbiota at various body sites (Table 1.3); or for clinicians, for example incubation periods of human pathogenic viruses (Table 7.3). The glossary at the end of the book provides short explanation of several terms that might be unfamiliar for some readers.

There are several drawbacks of this short textbook. The most important one is the absence of the references and bibliography for further reading. Although this is perhaps done to maintain the size of the textbook, this practice is rather unusual for a scientific textbook. The omission of suggested reading also hampers the interested readers to find good further reading. Another limitation of this book is that it does not follow the changes in taxonomy and terms. For example, it continues using the old concept of microbial flora, which today has been replaced by more specific terms, such microbiota. Lastly, the case studies that conclude many chapters in this book appear to be forced. It is a good idea to put the case study in clinical context and it is understandable that this part is included to satisfy students who want to use this book for preparation of United States Medical License Examination (USMLE). Yet, the cases are rather too short and no explanations accompanying the answers are given. By the same token, the practice questions in USMLE format, a special part at the end of the book, are in my opinion rather unnecessary. This additional learning part is more suitable for an online learning platform linked with the textbook, that in many other textbooks are provided by the publishers.

There is minimal change in the organization of the chapters in respect to the previous edition. Sherris sixth edition uses the classic approach where the introduction part is followed by four other parts focused on pathogenic viruses, pathogenic bacteria, pathogenic fungi, and pathogenic parasites. These five parts are further divided in 57 chapters. The chapters on infections of various organ systems that constituted the sixth part of the previous edition are now combined into a special section of the book. The Part I of the book explains the nature of infection and the infection agents, the immune response to infection and the principles of laboratory diagnosis of infectious diseases. In this part, there are also chapters on hot topics in medical microbiology: infection control and emergence and global spread of infection. Yet, these chapters are too brief: the asepsis overview in the operating room could be extended and the role of molecular diagnostics in the epidemiology of infectious disease could be discussed. A dedicated chapter on bioterrorism and travel-associated infections are perhaps missing in this Part I of this book.

Part II is dedicated on the major viral diseases and comprises of chapter 6–20. The viruses that are discussed in this part are respiratory viruses, viruses that causes childhood exanthems, poxviruses, enteroviruses (where the recent outbreak of enterovirus D68 has not found the way yet into this textbook), hepatitis viruses, herpesviruses, viruses of diarrhea, arthropod-borne viruses, rabies, retroviruses, and papilloma viruses. An interesting chapter of this part is the chapter on persistent viral infections of the central nervous system where an overview is given to diseases such as progressive multifocal leukoencephalopathy but also on prions disease. In the part II of this book, the tables on the classification of DNA and RNA viruses have been updated. For readers who do not spend daily professional activities in virus area, it is always difficult to memorize the classification of the virus family. Perhaps a table where the classification is based on virion structure is a better idea.

Part III is dedicated to the major bacterial diseases and comprises chapter 21–41. It is the largest part of this book. All major bacterial pathogens from Staphylococci, Streptococci, and Enteroccci to sprirochetes and Mycoplasma are discussed in this part. There is also a chapter dedicated to anaerobes. The last chapter on dental and periodontal infections of this Part III is a bit out of tune. In the previous edition this chapter was a part of clinical aspects of infection, the part that is now merged into a section on infectious diseases: syndromes and etiologies.

Part IV is dedicated to the major fungal diseases and comprises chapters 42 to 47. The organization of this part is classic: superficial fungi, opportunistic fungi and systemic fungal pathogens were discussed separately.

The rapidly evolving classification schemes for the protozoa have led to revision of several chapters in part V (chapter 48–57) on pathogenic parasites in this book. The chapter entitled Apicomplexa replaces the chapter entitled Sporozoa in the previous edition; and the chapter Sarcomastigophora–The Amebas replaces the chapter entitled Rhizopods.

Overall, this book is very well written and covers broad scope of human pathogens. Putting the above mentioned complaints on this book aside, this student-friendly book serves well as a point of introduction to the major pathogens described.

## Conflict of interest statement

The authors declare that the research was conducted in the absence of any commercial or financial relationships that could be construed as a potential conflict of interest.

